# Supplementary material for: Are carbon emissions trading and green financial instruments synergistic? -Comprehensive quantitative research based on content analysis
Source: PLoS One. 2024 Mar 7;19(3):e0298601. doi: 10.1371/journal.pone.0298601 (PMC10919601; doi:10.1371/journal.pone.0298601)
Supplement: S1 Data — (ZIP) [file pone.0298601.s001.zip › Data/地级市Z_F工作报告.zip!/░▓╗╒/║╧╖╩╩╨/└┤╫╘╣1⁄2╓┌║┼í╛┬φ┐╦╩2╛▌═°í┐_2017.docx]

# **2017年政府工作报告**

发布时间：2017-01-16 15:13

【字体：[大](http://www.hefei.gov.cn/public/1741/javascript:void(0);) [中](http://www.hefei.gov.cn/public/1741/javascript:void(0);) [小](http://www.hefei.gov.cn/public/1741/javascript:void(0);)】

各位代表：

  现在，我代表市人民政府向大会报告工作，请予审议，并请政协委员和其他列席人员提出意见。

  一、2016年工作回顾

  过去一年，在省委省政府和市委的坚强领导下，我们全面贯彻党的十八大和十八届三中、四中、五中、六中全会精神，认真学习习近平总书记系列重要讲话特别是视察安徽重要讲话精神，牢固树立五大发展理念，坚持稳中求进工作总基调，深入推进供给侧结构性改革，加快创新转型升级发展，锐意进取，扎实工作，较好完成了市十五届人大四次会议确定的各项目标任务，实现了“十三五”良好开局。

  ——综合实力实现跨越。预计，全市生产总值6200亿元、增长10%左右；财政收入完成1114.1亿元、增长11.4%；规模以上工业总产值首次突破万亿大关；固定资产投资超过6500亿元。合肥在全省经济首位度不断提高，辐射带动力日益增强。

  ——城市能级明显提升。国务院正式批复合肥城市总体规划，确立了长三角城市群副中心城市、国家重要的科研教育基地、现代制造业基地和综合交通枢纽的战略定位。公路、铁路、水路、空路、电路、气路、网路等一批重大基础设施发挥支撑作用，引江济淮工程开工建设，轨道交通1号线开通运营，合肥阔步迈入地铁时代。

  ——区域发展勇创一流。肥西县、肥东县、长丰县进入全国百强县，巢湖市入选全国中小城市创新创业百强县，庐江县荣获全国十佳生态休闲旅游名县。包河区、蜀山区入围全国综合实力百强区，庐阳区荣获全国最佳商业环境城区，瑶海区国家老工业基地搬迁改造试点取得突破。高新区位居国家级高新区综合排名第7，经开区工业总量位居国家级开发区第8，新站高新区获批国家产城融合示范区，合巢经开区加快打造国家级燃气轮机产业基地。

  ——创新驱动引领未来。合芜蚌国家自主创新示范区、合肥综合性国家科学中心获批建设。重大创新平台形成体系，创新成果密集涌现。世界首条量子保密通信网络“京沪干线”合肥至上海段率先开通。全社会研发投入占GDP比重达到3.1%、高于全省和全国平均水平，发明专利授权量增长35%，国家高新技术企业增至1186家，主要创新指标稳居全国省会城市“十强”。

  ——改革开放纵深推进。系统推进全面创新改革试验，168项改革协同展开，形成了63项重大制度性成果，推出了一批在全省乃至全国具有示范引领意义的先行先试之作，改革红利不断释放。深度融入国家“三大战略”，实现在更宽领域扩大开放、拓展合作，在更大范围配置资源、集聚要素。

  ——群众生活持续改善。城镇、农村居民人均可支配收入分别达到34800元、17200元，分别增长9%和9.5%；全市民生支出705.6亿元，占财政支出82.1%，“32+9”项民生工程全面完成；城镇新增就业23.2万人，超额完成全年任务；9.47万贫困人口实现脱贫、112个贫困村全部出列，脱贫攻坚首战告捷；防洪抗灾夺取全面胜利，水毁住房群众全部乔迁新居。

  一年来，我们主要做了以下工作：

  （一）聚焦前沿，科技创新跃升新高度。启动建设超导核聚变中心、量子信息国家实验室等重大工程平台。中科大先研院、清华大学公共安全院、合工大智能制造研究院、中科院技术创新工程院等协同创新平台步入良性运转，北航合肥科学城等开工建设。建成科技孵化器37家，新建重点实验室和工程（技术）研究中心、企业技术中心326家，高新技术产业实现增加值1300亿元、增长12%。国家“双创”示范城市建设成果丰硕，建成各类众创空间49家，其中国家级11家。启动“双引双培”四大计划，新增国家“千人计划”专家30人。

  （二）瞄准高端，产业转型迈出新步伐。加快新型显示、机器人2个国家战略性新兴产业区域集聚发展试点建设，新型显示成为全国面板产能最大、产业链最完整的产业基地。首批4个省级战略性新兴产业集聚发展基地绩效评估位居全省前列，生物医药和高端医疗器械、创意文化入选第二批省级基地，6个省级基地集聚重点企业406家、实现产值1500亿元以上。京东方10.5代线主体工程封顶，集成电路、太阳能光伏、新能源汽车产业迅速增长，战略性新兴产业实现增加值685亿元、增长13.5%。大力推进新一轮工业技术改造，完成技改投资1350亿元、占工业投资62%，规上工业实现增加值2270亿元、增长9.8%。大力推进现代服务业集聚区建设，滨湖国际金融后台服务基地入驻机构增至16家，万达文旅城等重大项目建成运营，研发设计、大数据等高技术服务业和电子商务、服务外包等新兴业态蓬勃发展，国际徽商大会、家博会、农交会、文博会等重大展会成功举办，获批全国旅游标准化示范城市。消费结构加快升级，社会消费品零售总额达到2440亿元、增长12%左右。

  （三）奋力攻坚，改革开放实现新突破。新增上市企业3家、新三板挂牌企业43家，直接融资2300亿元左右、增长30%以上。民营新安银行获批筹建。“营改增”试点全面推开。股权和分红激励试点企业达到170家。全面实施“五证合一”、“双证整合”，新登记各类市场主体12.2万户、增长33%。马钢（合肥）公司冶炼生产线整体关停，4886名职工全部得到妥善安置。建立市县两级一体化平台，公共资源市场交易规模突破2500亿元。推进分级诊疗、公立医院等改革，建立家庭医生签约服务机制。强力推进大招商，全年招商引资3500亿元，其中外商直接投资28.08亿美元、增长12%。开辟首条国际航空货运航线，“合新欧”班列实现常态化运行并开通首趟回程班列，水运港年吞吐量超过20万标箱，水果、冰鲜水产品进境指定口岸建成运行，空港保税物流中心（B型）开工建设，国际邮件互换局正式运营。中德（合肥）智慧产业园、“侨梦苑”获批设立，与俄罗斯乌法市结为友好城市。积极推动与长三角、长江中游城市群、长江沿岸城市联动发展，深入推进合肥上海双城合作，加快合肥都市圈一体化建设。扎实开展与皖北、皖西结对合作。援疆、援藏工作得到国家和省里肯定。

  （四）提升品质，城市建设彰显新形象。全面实施“1331”城市空间发展战略，新型城镇化、都市区国际化等重大规划加快编制。全年大建设新建、续建工程798项，完成投资421.1亿元、增长13.2%。合宁高铁等纳入国家中长期铁路规划，商合杭合肥段全面施工，合安客专加快建设，庐铜铁路基本建成；国省干线高标准建设；店埠河、合裕线航道升级改造基本完工。在轨道交通1号线建成的同时，2、3号线全面建设，4、5号线部分节点工程有序启动。庐州大道、南淝河路等加快改造。推进精品城市建设，一批特色街区形成品牌。地下综合管廊、海绵城市建设取得新进展。城市环境综合整治成效明显。126位市民入选“中国好人”，位居全国前列。

  （五）强化统筹，城乡联动形成新格局。推动五县（市）加快工业发展，县域经济占全市比重提高到33%左右。设立30亿元专项资金支持县（市）大建设，整合50亿元资金支持县（市）区社会事业发展和民生保障。粮食生产保持稳定，新建粮仓库容23万吨，市级以上龙头企业增至775家，市级以上现代农业示范区发展到32个。大力实施土地综合整治，耕地连续18年实现“占补平衡”。11条环巢湖美丽乡村示范带建设有序展开，完成34个乡镇政府驻地建成区整治和67个中心村建设。

农村道路畅通工程全面启动，开工建设1519个项目，建成通行2786公里。灾后水利水毁修复与水利建设有力推进，新解决22万农村人口饮水安全问题。

  （六）持续发力，生态环保取得新成绩。推进环巢湖生态修复保护工程，实施南淝河、十五里河、派河、双桥河等综合治理，建成投运蔡田铺、小仓房二期等污水处理厂，环巢湖35个乡镇污水处理厂全部运营，城市污水集中处理率达到98%，巢湖水质总体保持Ⅳ类。大气污染防治“九大行动”重拳出击，淘汰黄标车和老旧车近2.5万辆，全域全年全面秸秆禁烧成效显著，全市PM10浓度83微克/立方米、同比下降9.8%。“五森工程”和“绿化品质大提升”行动加快实施，新增人工造林14.2万亩、城镇绿化1710万平方米，创建省森林城镇7个、森林村庄71个，肥西三河进入国家级湿地公园建设试点。节能减排任务全面完成。

  （七）提标扩面，民生保障增添新福祉。建立健全“1+20+X”精准扶贫政策体系，在全省率先开展建档立卡贫困人口医疗补充商业保险试点和低保、五保、扶贫“三线合一”。加强就业培训和创业孵化，城镇登记失业率控制在3.03%。低保标准稳步提高，城乡基本养老、基本医疗保险、居民大病保险、被征地农民保障实现全覆盖。完善社会救助和保障标准与物价上涨联动机制。建成100个社区养老服务站、89个幸福院、23个农村敬老院“特护区”。出台房地产市场调控“十条新政”，房价过快上涨势头得到遏制。启动城中村危旧房改造项目10个，开工建设棚户区改造安置房3.49万套；实施35个老旧小区综合整治，1.13万户、3.76万群众受惠。学前教育三年行动计划如期完成，新建和改扩建中小学、幼儿园68所，创建121所市级新优质学校、50个市级名师工作室，完成492所农村学校“全面改薄”任务，市区中小学生午餐服务工程覆盖率达88.4%。公共文化服务设施网络和空间不断拓展完善，多次荣膺“中国十大最爱阅读城市”。《合肥通史》出版发行。推进健康合肥建设，启动一批医养结合示范项目。二孩政策全面实施，人口计生服务水平不断提升。全民健身蔚然成风，世界铁人三项赛、合肥国际马拉松赛等成功举办，形成环巢湖体育“水陆空”四季品牌赛事格局。

  （八）强化安全，社会治理提升新水平。平安合肥建设深入推进，“天网”工程一期建成，八类恶性案件发案数大幅下降。深入开展“铸安”行动，强化隐患排查整治，安全生产形势稳定向好。食品药品重大安全事件“零发生”。打击传销取得决定性胜利。“大信访”工作格局基本建立，信访渠道进一步畅通。荣获全国“六五”普法先进城市，城市法治建设评估位居全国第六。实现全国双拥模范城“八连冠”。突发事件应急处置能力得到加强，社区民防示范点建设全面推开，人民防空、防震减灾、残疾人工作成为全国先进城市。民族宗教、地方志、新闻、科普、气象、统计、档案、保密等工作水平实现新提升，工会、共青团、妇女儿童、红十字会、老年人和关心下一代等工作取得新进步。

  各位代表！

  过去一年，我们认真开展“两学一做”学习教育和“讲看齐、见行动”学习讨论，全面加强政府自身建设。强化依法行政，自觉接受人大监督，严格执行人大及其常委会的决议决定，主动接受政协民主监督、社会监督和舆论监督，全年办理人大代表议案和建议205件、政协提案631件。大力推进“放管服”改革，出台权力清单、责任清单动态调整和权力运行监督管理办法，行政审批事项和流程进一步精简优化。严格落实党风廉政建设责任制，持续强化行政监察和审计监督，坚决查处侵害群众利益的腐败问题。

  各位代表！

  过去一年，合肥发展稳中有进，稳中向好，争先进位，质量提升，成绩来之不易。这是省委省政府和市委坚强领导的结果，是市人大、市政协和社会各界大力支持的结果，是全市广大干部群众团结奋斗的结果。在此，我谨代表合肥市人民政府，向全市广大工人、农民、知识分子、干部、驻肥解放军指战员、武警官兵和政法干警，向各民主党派、工商联、人民团体和社会各界人士，向所有关心支持合肥建设和发展的海内外朋友，表示衷心的感谢，并致以崇高的敬意！

  在肯定成绩的同时，我们也清醒地看到当前发展中存在的问题和不足：经济下行压力仍然较大，部分行业和企业面临诸多困难；传统产业增长动力不足、转型不快；服务业新业态发展不快、总量不大；内陆经济开放高地没有形成；巢湖综合治理和大气污染防治任务艰巨；城市公共交通基础设施不能充分满足群众出行需要；教育、医疗、文化等基本公共服务均等化、优质化水平有待提升；征地拆迁、信访维稳等领域矛盾纠纷化解工作需要加强；少数部门和工作人员不作为、慢作为，能力不强、效率不高、作风不实、服务不优的问题依然存在。对此，我们将高度重视，采取切实有效措施加以解决。

  二、2017年主要任务

  当前，合肥发展正站在新的历史起点上，丰厚的科教资源为创新发展提供了有利条件，壮大的产业优势为转型升级奠定了坚实基础，提升的城市影响力为集聚要素营造了良好环境。中央统筹推进长江经济带建设等“三大战略”，省委省政府大力实施五大发展行动计划，为我们加快发展提供了重大机遇和广阔空间。合肥发展仍处于大有可为的战略机遇期，我们要认清形势、把握大局，坚定信心、乘势而上。

  今年是实施“十三五”规划的重要一年，也是本届政府的收官之年，做好今年工作意义重大。我们要把握新方位。经济新常态、发展新理念标注着我国经济新方位。在向着新愿景破浪前行的航程中，我们要进一步推进创新转型升级发展，加快培育新动能、蓄积新势能、催生新经济，积极抢占发展制高点。我们要找准新坐标。国家赋予合肥新的战略定位，省委省政府对合肥寄予厚望，我们要进一步登高望远、对标定向，放眼全局找准位置，在全省主动领跑、勇于率先，在全国积极追赶、勇于争先。我们要探寻新路径。开启发展新征程，我们要进一步解放思想，与时俱进拓展思路，坚持以改革突破制约，以创新占据先机，以实干赢得主动，在引领发展新常态中闯出新路子，在践行发展新理念中开辟新境界。我们要彰显新气质。面对复杂的宏观环境和繁重的发展任务，我们要进一步提振精气神，以坚守信仰的特质、中流击水的自信、力拔山兮的气概，不断赋予合肥清新大气、开放卓越的新气质。

  今年政府工作的总体要求是：全面贯彻党的十八大和十八届三中、四中、五中、六中全会精神，深入贯彻习近平总书记系列重要讲话特别是视察安徽重要讲话精神，认真贯彻省、市党代会精神，统筹推进“五位一体”总体布局，协调推进“四个全面”战略布局，牢固树立和贯彻落实新发展理念，坚持稳中求进工作总基调，以推进供给侧结构性改革为主线，以提高发展质量和效益为中心，大力实施五大发展行动计划，全面做好稳增长、促改革、调结构、惠民生、防风险各项工作，保持经济社会平稳健康较快发展。

  今年经济社会发展的预期目标是：全市生产总值增长9.5%左右；规模以上工业增加值增长9.5%左右；财政收入增长与经济增长保持同步；固定资产投资增长11.5%左右；社会消费品零售总额增长12%左右；居民人均可支配收入增长9%左右；城镇登记失业率控制在4.5%以内；居民消费价格涨幅控制在3%左右；单位生产总值能耗下降率及主要污染物减排量达到省控目标。

（一）推进创新发展，增强转型升级动能。

  大力实施创新驱动发展战略，深化供给侧结构性改革，推行“互联网+”模式，构建现代产业体系，在系统推进全面创新改革试验区和合芜蚌国家自主创新示范区建设中，充分发挥核心带动作用。

  提升自主创新能力。加快建设中科大高新园区、量子信息国家实验室与量子科技创新研究院，推进超导核聚变中心、天地一体化信息网络、离子医学中心、智慧能源创新中心等重大工程平台建设，全力打造合肥综合性国家科学中心和全国性产业创新中心。加快推动中科大先研院、北航合肥科学城等建设发展，完善提升协同创新平台功能。规划建设环巢湖科技创新走廊，加快建成安徽创新馆，形成面向全球的创新成果路演中心，打造全国重要的科技创新策源地和全省创新发展新引擎。集聚国内外高端创新服务机构，建设一批技术转移、知识产权服务、检验检测、科技成果转化交易等创新公共服务中心，争创国家科技成果转移转化示范区。新建企业工程（技术）研究中心、企业技术中心、重点（工程）实验室100户。推进国家“双创”示范城市和双创示范基地建设，打造一批全要素、开放式、便利化众创空间和特色小镇。突出“高精尖缺”导向，坚持“外引内培”并重，加快集聚一批具有世界水平的高端人才和创新团队，积极创建国家海外人才“离岸创业”基地。全年新增国家高新技术企业140户，高新技术产业增加值增长11%以上，全社会研发投入增长10%以上，发明专利授权量增长15%以上。

  推进供给侧结构性改革。积极引导产能过剩企业通过兼并重组等方式实现主动退出。全面落实各项税收优惠政策，开展“降价减费”行动，打好降低制度性交易成本、用能用地用工成本、物流成本等“组合拳”， 全方位服务好中小微企业，服务好实体经济发展。全面落实房地产市场调控“十条新政”，合理增加居住用地和住房供应，加快人才公寓建设，加强公租房分配和入住管理，强化市场联合监管和舆论引导，真正让“房子是用来住的，而不是用来炒的”，保持房地产市场持续健康发展。精准调整土地利用总体规划，合理布局项目新增建设用地。加强多层次资本市场体系建设，扩大直接融资比重，推动企业股权融资、上市融资。吸引内外资金融机构、功能性总部在肥落户，加快筹建民营新安银行，健全地方金融服务体系。大力发展天使投资、风险投资、产业基金，加快打造“基金丛林”。创新财政金融产品，落实“税融通”、“4321”政银担合作等政策。全面加强金融监管和政府债务管控，确保不发生系统性、区域性风险。深化国资国企改革，大力发展混合所有制经济，充分发挥建投、产投、兴泰等国资运营平台在推进企业改革重组、培育新兴产业等方面的引导作用。实施民营经济提升工程，修订促进民营经济发展条例实施细则，加大政策支持和服务保障力度，建立“亲”“清”新型政商关系，增强民营企业家信心。落实促进民间投资政策，加大PPP模式推广应用力度，推出一批示范合作项目。深化农业供给侧结构性改革，规范农村承包土地经营权流转，扩大农村集体资产股份制改革试点。

  构建现代产业体系。坚持“工业立市”战略不动摇，坚定不移支持实体经济发展，做大做强一批引领未来的产业集群，让“合肥制造”成为支撑合肥发展的坚强基石。以建设战略性新兴产业集聚发展基地为突破口，统筹推进“三重一创”，在集成电路、太阳能光伏、高端数控机床、新能源汽车、生物医药及高端医疗装备、燃气轮机、智能机器人、语音等重点领域聚焦发力，加快推动江淮大众新能源汽车、京东方10.5代线等一批重大项目建设。以智能化工厂改造为突破口，以家电、装备制造、汽车及零部件、食品加工等传统优势产业为重点，加快实施新一轮工业技术改造三年行动计划，全年完成工业技改投资1420亿元，积极争创“中国制造2025”示范城市。全面开展质量强市活动。以建设服务业集聚区为突破口，全面加快现代服务业发展。大力发展金融保险、现代物流、中介咨询等生产性服务业和大数据、工业设计等高技术服务业，积极创建中国软件名城。大力发展健康养老、旅游休闲等生活性服务业，做大做强会展业。大力建设环巢湖国家旅游休闲区，推进万达文旅城二期等重大项目建设，支持中庙·姥山岛创建国家5A级景区、半汤创建国家级旅游度假区。加快商圈布局优化和升级改造，打造精品特色商业街区，争创国家级中央商务区。

  （二）推进协调发展，放大区域联动格局。

  强化省会城市意识，在全国积极推动区域互动发展，在全省主动加强与其他城市联动发展，不断增强中心城市的辐射带动力。

  加强区域联动发展。全面融入长三角，在交通设施、产业发展、金融服务、科技创新、人才交流等方面，深化与沪宁杭等城市联动发展，深入推动合肥上海双城合作。聚焦全国性综合交通枢纽建设，加快商合杭、合安客专等高铁建设，启动合宁、合六、合马等城际铁路建设前期工作。加强与珠三角、京津冀等区域交流互动，积极承接北京非首都核心功能疏解。推进与长江中游城市群互联互通。推动合肥都市圈扩容升级，与芜湖、马鞍山跨江联动，与宁杭等都市圈对接互动，完善合作机制，优化产业分工，推进基础设施建设一体化。加强与皖北、皖西结对合作。

  推进城乡一体化发展。继续设立30亿元专项资金支持县（市）大建设，整合50亿元资金支持县（市）区社会事业发展和民生保障。实施新型城镇化规划，加强城乡规划和建设标准衔接，推动市政基础设施延伸，全面提高县域基础设施建设水平。推进农村道路畅通工程建设三年计划，全年建成通车5000公里以上。开展农村客运公交化改造，加快城乡公交换乘枢纽建设。开工建设环巢湖防洪治理等工程，全面完成水毁水利设施修复任务。大力实施农村土地综合整治，补充耕地数量，提高耕地质量。加强社会保障城乡衔接和社会事业城乡对接，加快城乡公共服务均等化进程。推进乡镇建成区以及中心村整治建设，支持巢湖市开展美丽乡村整市创建试点，高水平规划建设环湖十二镇，加快构建环巢湖美丽乡村示范带。

  加快县域经济发展。坚持走新型工业化道路，大力实施工业强县战略，进一步提升县域经济发展质量和层次。完善县域工业园区基础设施，推进标准化厂房建设，增强产业承载力和综合服务功能。优化县域工业布局，推进园区整合和扩容升级，推动开发区、城区都市产业园与县域工业园区合作共建一批大项目。优化县域产业结构，加快商贸、物流、旅游等服务业发展。合理确定乡镇功能定位，完善分类考核评价办法。肥西、肥东、长丰三县要加快与中心城区等高对接、融合发展，巩固提升全国百强县位次；巢湖市要彰显湖光山色特色品质，加快建成一座靓丽的明珠之城，庐江县要加快副中心建设，争创生态宜居、城乡统筹的新典范，共同冲刺全国百强县。

  大力发展都市型现代农业。推进农业多功能开发，加快都市休闲农业等新兴业态发展，推动一二三产业深度融合。实施“粮安工程”，稳定粮食产能，强化“菜篮子”安全供给保障，提升畜水产品供给能力。大力发展现代农业产业化联合体，培育壮大家庭农场、农民合作社、农业产业化龙头企业，新增各类新型农业经营主体1000家以上。实施农业科技创新提升行动和农机化提升工程，加强现代农业园区建设，推动设施农业提档升级。完成全国第三次农业普查任务。加快现代农业气象示范基地建设，加大农业气象适用技术推广力度。加快“三品一标”优质农产品扩面增量，严格农产品质量安全监管。

  提高城市建设品质。争取国家级合肥滨湖新区尽快获批，以“创新+生态”为主题，高起点规划，高标准建设。加快新合肥西站、高铁南站南广场等重点项目建设。谋划推动骆岗通用机场、滨湖水上机场枢纽建设。开工建设新合蚌路等工程，进一步优化国省干线路网结构。加快丰乐河航道升级改造，启动派河港码头一期。积极发展地铁经济，创新轨道交通建设和管理运营模式，提高建设速度和运营效率，确保2号线下半年建成通车，3、4、5号线加快建设。完善城市路网结构，建成上海路、绕城高速改线等工程，推进阜阳北路高架北延、裕溪路高架东延、长江西路快速化改造等建设。加强城市地下综合管廊和海绵城市建设，提升水电气热等市政设施保障能力。启动长江中路街区改造工程，实施老城隍庙片区、淝河片区等改造，突出老城区特色风貌和合肥历史记忆，整体打造一批有品位、有内涵、“最合肥”的文化街区。瑶海、庐阳、蜀山、包河四个城区要加快转型发展，完善城市功能，全力打造国际化都市区核心区、高端服务业集聚区、精品特色先行区和全省“辐射源”。

加强城市综合治理。全面开展城市环境综合整治，进一步优化城市人居环境。深入创建“公交都市”，优化提升公交路线覆盖广度、运行效率和通达深度，加快形成轨道交通、快速公交、普通公交于一体的城市综合客运网络。完善城市慢行系统，在老城区、高校园区、开发区、轨道交通线周边区等开展公共自行车服务试点，推动多种交通工具“零接驳”、“零换乘”。加强城区公共停车场建设，新建泊位1万个以上。加大交通管理执法力度，优化重点区域交通组织，提升交通治理法治化、精细化水平。坚持一街一特色，加强城市立面整治，实施夜景亮化工程，突出 “水、绿、城、桥”等元素，提升城市夜间文化品位。深入开展老旧小区综合整治、城中村和老旧小区搬迁改造，全面提升住宅小区物业管理水平，加快形成社区15分钟生活圈。强化基于云计算的大数据开发与利用，加快构建智慧城市、信用城市体系。深化城市执法体制和街道社区管理体制改革，完善“数字城管”运行机制，探索开展“小街区规制”试点。

  （三）推进绿色发展，厚植生态文明优势。

  牢固树立青山绿水就是金山银山的理念，以国家级巢湖生态文明先行示范区建设为统揽，打好水、气、土污染治理“三大战役”，全域打造美丽合肥。

  创造大湖治理样板。积极推进引江济淮工程合肥段建设，加快实施环巢湖综合治理工程。强化城市水环境整治，对城市防洪、污水处理、供水保障、水岸景观等实行综合施治，重点加强南淝河、十五里河、派河、双桥河等综合治理，加快建成十五里河污水处理厂三期、许小河及关镇河生态补水、清溪路净水厂配套管网等工程，提升环巢湖乡镇污水处理厂运营监管水平。加大治理力度，基本消除城市黑臭水体。加快环巢湖农业面源污染综合防治实验区建设，推进化肥和农药减量化使用。

  创建国家生态园林城市。持续开展“绿化品质大提升”行动，着力构建襟江滨湖的大生态、大绿化格局。突出打造环巢湖大道、滁河干渠等沿线绿色风光带，强化外环高速等绿化廊道建设，抓好沿派河大道等省级示范段创建，推进柏堰湖、王咀湖、少荃湖等片区生态修复和综合整治，加快肥西三河、庐阳董铺、巢湖半岛等国家级湿地公园试点建设，新建森林长廊228.8公里。以扩绿造景、增花添彩为主线，完成城镇园林绿化新增、改造面积960万平方米以上，新建庐州公园二期、四季花海C区等公园游园60个以上，进一步提升公园及绿地服务半径覆盖率。统筹推进农村道路、河渠、水库、塘坝、庭院等绿化，完成成片造林5.4万亩。

  强化重点领域污染防治。加强雾霾治理，重点推进工业“三废”治理，实施燃煤电厂超低排放改造，提升挥发性有机物整治标准，完成禁燃区燃煤锅炉淘汰任务；加强机动车路检、抽检和遥感监测，开展高残值黄标车技改；严控道路交通、施工工地、料场、裸露地面等扬尘污染，推进建筑垃圾、工程渣土全密闭运输；扎实开展全域全面全年秸秆禁烧，大力提高秸秆综合利用水平。加大主城区烟花爆竹禁放管理力度。实施重点路段降噪工程，有效治理噪声污染。加强餐厨废弃物资源化利用，完善固体废弃物收运处置系统，加快龙泉山垃圾处理场生态修复建设。推进矿山整治与修复。加强土壤污染防治，加快原红四方、马钢（合肥）公司等地块土壤污染治理修复。实施循环发展引领计划，强化工业园区循环化改造，加快建立再生资源回收利用体系。

  健全生态文明制度。完善百名河长治百河的“河长制”，实行最严格的责任分工和考核奖惩。开展节能减碳全民行动，强化工业、交通、建筑、公共机构等领域节能减排，加快低碳城市建设。深入推进节约集约用地试点市建设，全面创建节水型社会。严格“三线一清单”约束，全面推行排污许可证制度，探索建立排污权交易和生态补偿机制，落实污染联防联控机制。

  （四）推进开放发展，构筑对外开放高地。

  实施大开放战略，充分发挥“一带一路”、长江经济带双节点城市功能，拓展通道、提升平台、优化环境，加快打造开放新高地和国际化都市区。

  提升开放平台。积极争创国家自贸区。规划建设国际内陆港，加快申报国家铁路开放口岸，不断提升“合新欧”班列运行能力；建成启用航空港食用水生动物、药品进境指定口岸，努力开辟国际航空货运新航线；大力引进国际干线船公司和货代船代企业，推动水运港吞吐量稳定增长。加快综合保税区重大项目建设，推进出口加工区争创综合保税区，建成空港保税物流中心（B型），提高跨境电商综合试验区建设水平。培育外贸增长新动能，促进外贸回稳向好。按照对外开放先行区、创新创业引领区、新兴产业集聚区的定位，高新区要在合芜蚌国家自主创新示范区建设中当好龙头，经开区要在创新转型升级发展中率先突破，新站高新区要在创新发展上提升总量，合巢经开区要在高端产业培育发展上奋力争先。

  拓展国际合作。加强与“一带一路”沿线国家和地区务实合作，推动汽车、家电、新能源、装备制造等优势产能和产品“走出去”。加快中德（合肥）智慧产业园、中德（合肥）智能制造国际创新园、“侨梦苑”等建设，打造一批高端国际合作平台。鼓励优势企业到境外资本市场上市融资、开展并购，加快培育一批具有国际竞争力的本土跨国公司。推进国际友城交流，深化科技、教育、文化、体育、旅游等领域合作，积极争取承办专业化、高端化的国际会议、国际论坛。

  优化开放环境。依托海关特殊监管区域，全面推广一站式作业和关检合作“三个一”等新型通关模式。加快建设国际学校、国际医院、国际社区、国际文化演艺场馆等服务设施，开展公共场所国际化通用标识体系建设。加强在肥境外人员服务管理，落实口岸签证政策。完善现有地方性法规和规章，加快形成与国际投资、国际贸易通行规则相衔接的制度体系。

  开展精准招商。进一步完善大项目招商引资政策导则，突出抓好战略性新兴产业、现代服务业招商引资，积极开展集成电路、大数据、智能制造等专题招商。继续开展“县干大招商”等活动，依托“合肥之友”、商会协会等平台，紧盯长三角、珠三角、京津冀等重点区域，深化与世界500强、中国500强企业以及央企、知名民企合作，大力引进重大台资项目。突出招大引强，注重招才引智，优化招商结构，提升招商水平。全年招商引资3850亿元，利用外资增长7.5%。

  （五）推进共享发展，提升群众幸福指数。

  加快公共产品和公共服务均等化、优质化，让人民群众物质更富足、精神更富有、生活更幸福，切实增强民生保障的感受度和获得感。

  全力保障民生。完善就业创业扶持政策体系，健全就业援助制度，构建大众创业与扩大就业良性互动机制。深入推进青年创业计划，加快青年创业园等创业孵化基地建设。完善项目选择与退出机制，加强绩效评估管理，充分发挥民生工程的惠民作用。实施全民参保计划，加快推进机关事业单位养老保险参保工作，做好城乡养老保险制度改革及制度衔接服务，推进城镇职工和城镇居民大病医疗保险。完善低保政策，加大对城乡低保、特困人员救助力度。实施居家养老服务条例，打造一批示范养老社区。大力推进脱贫攻坚行动计划，全面完成脱贫任务，建立和完善监测评估、返贫预警机制，探索制定低收入群体扶持政策，确保全面小康路上不落一人。

坚持教育优先。加大教育投入，大力发展公办幼儿园，积极发展普惠幼儿园，多建群众上得起的幼儿园，城区建成使用幼儿园24所，新开工建设51所。继续实施义务教育“三大提升工程”，切实提高农村师资水平，加快优质教育均衡化，实施消除大额班计划，提升中小学生午餐服务工程城乡覆盖率，加快推进合肥新四中、市青少年综合实践基地、市特教中心北校区建设和巢湖二中迁建，城区建成使用中小学25所，新开工建设35所。全面创建美丽校园，办好老百姓家门口的学校，打造教育名城。实施职业教育质量提升工程，推动产教融合、校企合作、国际合作，组建合肥技师学院，推进黄麓师范学校改扩建工程，建成市职业教育公共实训基地，支持合肥职业技术学院、合肥幼儿师专建设地方技能型高水平大学，打造职教高地。支持中科大建设世界一流大学，支持合工大、安大等建设一流学科专业和高水平大学，加快中德教育合作示范基地建设，支持合肥学院建设地方应用型高水平大学，打造大学之城。

  建设健康合肥。深化医药卫生体制综合改革，加强“三医联动”，实现多层次医疗服务体系、重点人群家庭医生签约服务、重点人群医疗救助、多元化健康服务等“四个全覆盖”。加大医疗卫生投入，优化资源配置，加快实施市公共卫生管理中心、市一院门急诊住院综合楼、市三院整体搬迁等重点项目，启动居民健康卡工程。推进医养结合，加快建设市一院、二院老年护理院等示范项目。坚持计划生育基本国策，落实全面二孩政策，促进人口均衡发展。加强健身场地设施规划建设，加快构建10分钟健身圈，办好老百姓家门口的健身工程。广泛开展青春毅行等全民健身活动，进一步打造世界铁人三项赛、合肥国际马拉松赛等环巢湖体育品牌赛事。

  推进文化强市。深入践行社会主义核心价值体系，全面开展公共文明引导行动，扎实推进文明单位、文明村镇、文明家庭等创建活动，加强公民思想道德建设，注重家庭、家风、家教建设，不断提升市民文明素质，打造精神文明创建“升级版”。倡导科学精神，大力实施全民科学素质行动计划。加强公共文化服务体系建设，规划建设市中心图书馆、美术馆、科技馆新馆、自然博物馆、青少年活动中心、合肥广播电视塔等重大文化工程，建成市少儿图书馆过渡馆。完善政府向社会力量购买公共文化服务机制，推动公共文化设施社会化运营。深入开展“大湖名城、悦读合肥”全民阅读活动，建设一批城市阅读空间，打造“书香合肥”。推动各县（市）区“两馆一场”达标升级，大力建设乡镇、社区综合文化服务中心示范点和中心村农民文化乐园。扶持优秀文化产品创作生产，积极举办全国性文化活动，推出一批具有全国影响力的文化品牌和文艺精品。加强文化遗产传承保护和合理利用，振兴庐剧、巢湖民歌等传统文化艺术。实施重大项目带动战略，大力推进文化和科技融合、数字出版等国家级文化产业基地建设，加快发展文化旅游、动漫游戏、文化创意等新兴文化业态。

  创新社会治理。启动建设一批社区中心、邻里中心，提升社区管理服务功能。加快完善公共法律服务体系，深入开展“七五”普法。推进全国公共安全城市试点建设，建立立体化信息化社会治安防控体系。完善“三调联动”、矛盾纠纷排查化解、社会稳定风险评估、领导干部接访下访、积案化解、领导包案等工作机制，织密织牢公共安全网。加强互联网安全管理，构建和谐文明网络空间。强化防空防灾设施建设，深入推进民防进社区。完善应急管理体系，提升突发事件处置实战能力。健全安全监管体制机制，积极开展安全生产执法年活动，强化企业主体责任，严防较大以上事故发生。加强食品药品安全监管，积极创建国家食品安全示范城市。保持高压态势，严防传销回潮。加强国防和后备力量建设，提升全国双拥模范城“八连冠”成果，支持深化国防和军队改革，争创国家军民融合示范城市。支持工会、共青团、妇联、红十字会等人民团体广泛参与社会管理和公共服务，扎实做好民族宗教、防震减灾、地方志、新闻、档案、保密、残疾人等工作。

  三、加强政府自身建设

  新形势新任务对加强政府自身建设提出了新的更高要求，我们要全面贯彻新发展理念，大力提升行政效能，进一步增强执行力和公信力，努力打造人民满意政府。

  强化看齐意识，做到政治坚定。牢固树立政治意识、大局意识、核心意识、看齐意识，特别是核心意识、看齐意识，始终与以习近平同志为核心的党中央方向一致、步调一致，始终做党的理论和路线方针政策的忠诚捍卫者、模范执行者。深入开展“讲看齐、见行动”活动，使学习讨论的过程成为坚定信仰、成就理想的过程，成为解放思想、创新思路的过程，成为提高素质、增长本领的过程，成为推动工作、造福人民的过程。

  强化法治意识，做到依法行政。忠于宪法，忠于法律，坚持依法行政。自觉接受人大监督，严格执行人大及其常委会决议决定，认真办理人大代表议案和建议。主动接受政协民主监督，广泛听取民主党派、工商联、无党派人士、人民团体和社会各界的意见，高度重视社会监督和舆论监督。出台法治政府建设实施方案，健全科学民主决策制度，建立“决策、督查、评估”三位一体工作机制，努力营造法治化、市场化、国际化的政务环境，让权力在阳光下运行。

  强化担当意识，做到奋发有为。牢固树立以人民为中心的发展思想，把担当负责的精神落实到实际工作中。努力营造鼓励干事创业的政治生态，旗帜鲜明地为敢担当的干部勇担责，让敢负责的干部有奔头，让干成事的干部有前程，引导广大公务员多一份只争朝夕的紧迫感，多一份勇挑重担的使命感，多一份服务大众的光荣感，撸起袖子加油干，共创合肥新未来。

  强化服务意识，做到简政敬民。把群众当作亲人，为民服务做到“温、良、恭、勤”。以善良之心对待百姓，把党和政府的温暖送到千家万户。以谦恭之心躬身问民，聆听群众最深处的声音，为民解忧更接地气，为民办事更具质感。以敬民之心行简政之道，深化“放管服”改革，简化审批事项，优化审批流程，把方便留给群众。以勤勉之心履职尽责，全面掀起新一轮效能革命，强化监督问责，坚决整治庸懒散慢，推动政府工作提质增效。

  强化廉政意识，做到风清气正。把干事作为最永恒的追求，把干净作为最起码的准则，始终把纪律和规矩挺在前面，自觉践行“三严三实”要求，做到守底线、知荣辱、明大德。认真落实党风廉政建设“两个责任”，筑牢思想防线，扎紧制度笼子，坚决以零容忍态度惩治腐败。全面落实中央“八项规定”、国务院“约法三章”和省市各项规定，加强行政监察和审计监督，持之以恒抓作风、整“四风”， 努力实现干部清正、政府清廉、政治清明。

  各位代表！

  梦想照亮前方，奋斗赢得未来。让我们紧密团结在以习近平同志为核心的党中央周围，在省委省政府和市委的坚强领导下，开拓创新，攻坚克难，加快建设长三角世界级城市群副中心和具有国际影响力的创新之都，进一步开创“大湖名城、创新高地”建设新局面，以优异成绩迎接党的十九大胜利召开！
